# Three-Dimensional Human Cell Culture Models to Study the Pathophysiology of the Anterior Eye

**DOI:** 10.3390/pharmaceutics12121215

**Published:** 2020-12-15

**Authors:** Laura García-Posadas, Yolanda Diebold

**Affiliations:** 1 Instituto de Oftalmobiología Aplicada (IOBA), Universidad de Valladolid, 47011 Valladolid, Spain; yol@ioba.med.uva.es; 2 Centro de Investigación Biomédica en Red de Bioingeniería, Biomateriales y Nanomedicina (CIBER-BBN), Instituto de Salud Carlos III, 28029 Madrid, Spain

**Keywords:** three-dimensional models, anterior eye, ocular surface, in vitro, cell culture, biomaterials

## Abstract

In recent decades, the establishment of complex three-dimensional (3D) models of tissues has allowed researchers to perform high-quality studies and to not only advance knowledge of the physiology of these tissues but also mimic pathological conditions to test novel therapeutic strategies. The main advantage of 3D models is that they recapitulate the spatial architecture of tissues and thereby provide more physiologically relevant information. The eye is an extremely complex organ that comprises a large variety of highly heterogeneous tissues that are divided into two asymmetrical portions: the anterior and posterior segments. The anterior segment consists of the cornea, conjunctiva, iris, ciliary body, sclera, aqueous humor, and the lens. Different diseases in these tissues can have devastating effects. To study these pathologies and develop new treatments, the use of cell culture models is instrumental, and the better the model, the more relevant the results. Thus, the development of sophisticated 3D models of ocular tissues is a significant challenge with enormous potential. In this review, we present a comprehensive overview of the latest advances in the development of 3D in vitro models of the anterior segment of the eye, with a special focus on those that use human primary cells.

## 1. Introduction

The goal of biomedical research is to understand the biological processes that underlie diseases toward developing effective treatments. In the various pathologies that affect human and non-human animals, genes may be disrupted, proteins may be missing or, on the contrary, overexpressed, and signaling pathways may be altered. All of these mechanisms need to be studied in order to characterize the disease and identify therapeutic targets. At a later stage, during the development of a potential therapy, its safety, efficacy, and delivery, among many other parameters, must be studied in the laboratory before it reaches patients.

To perform these analyses, in vitro models are crucial. In vitro models help to fill the gap between the understanding of clinical evidence of disease and the development of complex, relevant in vivo models in which novel therapies can be tested. The physiological relevance of in vitro models is paramount for reaching the clinical step quickly and safely. According to Abbot and Kaplan, physiological relevance “is the characteristic of (or corresponding to) healthy or normal biological functioning” [1]. As reported by these authors, physiological relevance may vary in different contexts but, in general, it involves recapitulating the biological context (different cell types, extracellular matrix, vascularization), incorporating spatial cues (culturing cells in three dimensions), and including chemical and mechanical cues. Therefore, the development of in vitro models that are effective is the central issue. Key aspects include model complexity and the cell types used.

### 1.1. Increasing Complexity of In Vitro Models

Several decades ago, in vitro studies were typically performed in very oversimplified cell culture models. Cell culturing started with tissue culture in the late XIX century, when Wilhelm Roux demonstrated that it was possible to maintain living cells of a chicken embryo outside the body [2]. At the beginning of the XX century, Ross Harrison and Leo Loeb maintained live tissues or organ pieces in tubes. In addition, Harrison initiated the hanging drop technique [3]. Subsequently, Alexis Carrel and Montrose Burrows used this technique to grow tissues on plasma and were able to transfer the cells that migrated from the tissue [4]. An important feature of this technique was the use of trypsin, an enzyme that digests the bonds between cells, allowing them to be harvested and transferred to different culture surfaces. Carrel also established both the aseptic technique for cell culture and the first cell line [2,5]. Since then, extensive efforts have been made to optimize the culture conditions and culture media used to keep cells alive and proliferating in the laboratory. Cell culture systems continued to increase in their complexity, and an important milestone was reached with the progression from two-dimensional (2D) cell monolayers to three-dimensional (3D) cell cultures, supported by the early studies of Mina Bissell in the 1980s. Three-dimensional cell cultures create an artificial environment in which cells are able to interact and mimic the behaviors of the living organism from which they are derived. Thus, these culture systems provide more physiologically relevant information [6]. With increased knowledge about improved cell culture practices, new biomaterial properties, and tissue engineering techniques established in the last 30 years, the number of 3D models has significantly increased. Currently, 3D models are widely used in different disciplines, such as drug discovery and development [7,8,9,10,11].

The aim of 3D models is to recapitulate the spatial structure of tissues. Most tissues are composed of different layers of cells that are polarized, a biological property that is essential in tissue functioning. Therefore, a simple cell monolayer cannot represent the complexity of the whole tissue. Multilayered models of a single cell type are the simplest 3D models that, nevertheless, are able to produce more reliable results than monolayer cultures since cells in 3D models behave more similarly to those under natural conditions [12]. Spheroids are “spherical, heterogeneous aggregates of proliferating, quiescent, and necrotic cells in culture that retain 3D architecture and tissue-specific functions” [13]. Spheroids can serve as models by themselves or can be used as “building blocks” in tissue engineering [14]. A step forward in the development of 3D models is the combination of different cell types. Coculture procedures can be performed with different strategies: cells can directly contact each other, or special inserts can be used to keep them physically separated. All of these approaches allow researchers to study cell–cell interactions. In addition to the combination of different cell types, 3D models can also include the noncellular parts of tissues; these are the components of stroma, such as collagen, fibronectin, and laminin. Finally, the most complex models may also include the vascularization and/or innervation that characterize the modeled tissue.

In vitro 3D models can be developed using multiple techniques, one of which is 3D bioprinting. Three-dimensional bioprinting is a form of tissue engineering that was made possible by advances in 3D printing technologies. This technique allows for the precise positioning of biomaterials and living cells to create 3D structures [15]. Another alternative that deserves special attention is organ-on-a-chip, which is a combination of cells with 3D structures that are intended to represent realistic models of organs. The idea is not to produce an exact tissue equivalent or to build a complete living organ but to establish a functional unit that recapitulates certain aspects of the physiology in a simplified but precise manner [16].

Thus, there is a wide variety of 3D experimental models that are able to represent what happens in the live organism with much higher fidelity than classical 2D cell culture models [17]. That being said, it is important to note that, currently, 3D in vitro models typically lack sufficient complexity to completely replace the use of laboratory animals, but they can significantly reduce the number of these animals used in experimentation [18].

### 1.2. Cell Types Used in In Vitro Models

In vitro cell culture models, regardless of their complexity or the techniques used to create them, can be developed using primary cultures or immortalized cells, also known as cell lines. Both of these cell types can originate from humans or animals. Thus, the variety of cell-based models is extremely wide.

Cell lines are the most popular yet simplistic means of preparing in vitro models. They are widely available, easy to manage, and can be continuously passaged, allowing for the obtention of an almost unlimited number of cells [19,20]. As a result, it is easy to plan and perform experiments in a cost-effective manner. However, despite its benefits, cell lines have important disadvantages. First is the problem of cross-contamination. Several reports have shown that a large number of commonly used cell lines are not really the cells that researchers assume them to be [21,22,23], and cell lines used in vision research are no exception [24]. In addition, continued passaging results in phenotypic and genetic drifts that lead to nonreproducible and physiologically irrelevant results [25,26,27]. For this reason, primary cultures are often regarded as a better choice. However, primary cells, which are obtained directly from living tissues through biopsies or from cadaveric donors, are more difficult to obtain and culture, and they can only be maintained in vitro for a short period of time, rendering the research more time-consuming and complex. Furthermore, the obtention of tissue from biopsies is usually limited by ethics-related issues.

The cells used in different in vitro models can be obtained from humans or animals. The difficulties in obtaining human tissues have resulted in mice, rats, rabbits, and pigs being recurrent sources of biological material for research. However, interspecies differences may lead to erroneous interpretation of results. Consequently, in vitro models that use human primary cells are still more desirable because they are closer to the real scenario of human disease being studied.

### 1.3. In Vitro Models in Ophthalmic Research

**Figure 1:**
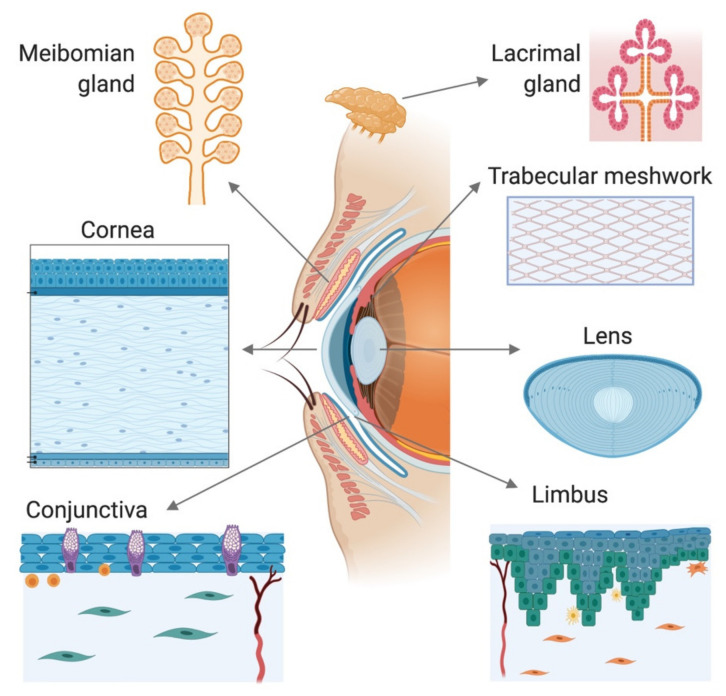
Anterior segment of the eye. Schematic showing the different tissues and structures that form the anterior eye. Basic histological structures of main tissues are also represented. Created with BioRender.com.

The eye is an extremely complex organ composed of a large variety of highly heterogeneous tissues. It can be divided into two asymmetrical segments that are separated by the lens. The anterior segment of the eye consists of the cornea, conjunctiva, iris, ciliary body, anterior portion of the sclera, aqueous humor, and the lens itself (Figure 1). The posterior segment, much larger in size, consists of the sclera, choroid, retina, and vitreous body [28].

To maintain eyesight, different ocular tissues are subjected to extremely tight regulation, and each one has very specific and different properties. Of these tissues, the cornea and the retina are the most studied.

Many different models are used to study the eye and to test drugs that are intended to treat ophthalmic diseases [29,30]. Since the number of models is too large to include in one manuscript, the aim of this review is to provide a comprehensive overview of the latest advances in the development of 3D in vitro models of the anterior segment of the eye, with a special focus on models that use human primary cells.

## 2. Three-Dimensional In Vitro Models of Anterior Eye Tissues

The anterior segment of the eye is composed of different tissues, each of which has different requirements. For this reason, these tissues are usually modeled separately. When developing a new model of tissue, the primary objective is to faithfully represent its key functional properties. In the next sections, we summarize the main characteristics of each tissue that constitutes the anterior eye and review the most significant models developed for their study.

Some structures of the anterior eye, such as the cornea and the lens, share common optical properties such as transparency, which facilitates the passage of light to the retina. Other structures, such as the conjunctiva and the limbus, possess an extraordinary functional complexity to support the maintenance of specific features in related structures, such as the transparency of the cornea. Similarly, tissues such as the trabecular meshwork, lens, and cornea have important biomechanical properties that allow resisting deformation and maintaining optical stability, and these are reliant on regional specializations of the connective tissue organization. All of these tissue-related intricacies make the development of 3D models a difficult challenge. In this section, we present an overview of the different strategies reported in the literature that address these challenges.

### 2.1. Tear Film and Its Production Tissues

Although not a tissue, tear film is an important component of the ocular surface. It is composed of water, electrolytes, and proteins that are produced by the main and accessory lacrimal glands; the mucins secreted by the lacrimal gland, corneal, and conjunctival epithelial cells (primarily by conjunctival goblet cells); and the lipids produced by meibomian glands [31]. This film moisturizes and protects ocular surface tissues. It acts as a barrier to the entrance of pathogens into the cornea, but a shortcoming of this function is that it also prevents topical drug administration. Thus, it is important to consider the effect of the tear film when evaluating the penetration of drugs through the cornea. Cell culture models representing goblet cells, an essential part of conjunctival epithelium, are reviewed in Section 2.4. Below, we review 3D models of lacrimal and meibomian glands.

#### 2.1.1. Lacrimal Gland

The lacrimal gland is an exocrine gland composed of multiple acini organized in lobules and ducts and myoepithelial cells that surround the acinar cells. Acinar epithelial cells secrete water and tear proteins. Afterward, this fluid passes through the ducts, where its electrolyte composition is modified [32]. Finally, it arrives at the ocular surface, where it mixes with the other components of the tear film: mucins and lipids.

The regeneration of the lacrimal gland has been proposed as a physiological, potentially long-lasting solution for the treatment of aqueous-deficient dry eye. To this end, different tissue-engineered equivalents and ex vivo models that can be used to reconstruct damaged lacrimal glands have been developed. These models were extensively reviewed by Liu et al. [33].

Primary cultures of the human lacrimal gland have recently been established [34]. In 2000, Yoshino created a 3D human lacrimal gland epithelial culture system using Matrigel^®^ and type I collagen gel with or without fibroblasts [35]. Different authors demonstrated that Matrigel^®^ contains extracellular matrix components that facilitate the development of a more complex 3D architecture in terms of dimensions and cell-to-cell contact compared with the conventional 2D culture system. Cells grown in Matrigel^®^ containing fibroblasts formed tubuloacinar structures that resembled the human lacrimal gland in vivo. Similar results were obtained with the lacrimal glands of mice using gelatin gels [36] and laminin 111 gel or Matrigel^®^ [37]. These results highlight the importance of 3D structures and cocultures in faithfully representing in vivo conditions.

The spheroid technique has also been applied to lacrimal gland cell culture using rabbit [38] and human [39] cells. In the latter study, Tiwari et al. isolated cells from fresh human lacrimal glands that were harvested from patients undergoing exenteration. The cells were then cultured in serum-free medium for 14 days in ultra-low attachment plates to form spheroids, which the authors called lacrispheres [39]. These lacrispheres were enriched in stem cells and retained secretory capacity, thus representing a promising option for lacrimal gland regeneration and a potential 3D model for in vitro research.

In summary, different methodologies have been used to establish 3D cultures of both animal and human lacrimal glands. Most of these bioengineered glands were prepared with the purpose of being transplanted and repairing impaired lacrimal glands. Nevertheless, they can be valuable if used as in vitro models to test drugs or study the pathophysiology of the tissue.

#### 2.1.2. Meibomian Glands

Meibomian glands are a modified type of sebaceous gland found within the eyelids. Their oily secretions form the lipid layer of the tear film, and they are essential for the maintenance of a healthy ocular surface. Indeed, meibomian gland dysfunction is one of the leading causes of dry eye disease [40]. Meibomian glands are arranged in parallel and are thought to work coordinately. Each gland is approximately linear and composed of clusters of acini organized around a central duct. The acinar cells, also called meibocytes, are able to synthesize lipids that are secreted to the tear film.

The development of meibography in the late 1970s and recent improvements in the technique have allowed for important in vivo evaluations in humans [41]. However, many studies still need to be performed in in vitro models of meibomian glands.

Cell cultures of meibomian glands have been established. David Sullivan’s group published a method to isolate viable human meibomian gland epithelial cells and to culture them in serum-free media [42]. They also immortalized these cells by introducing hTERT, creating the first (and, to date, only) human meibomian gland epithelial cell line (HMGEC). The cells maintain a cobblestone morphology and synthesize lipids that are identifiable with Oil Red O staining. Exposing HMGECs to serum promotes lipid production [43]. The authors who observed this suggested these cells be used as a preclinical model to develop new therapeutic strategies for treating meibomian gland dysfunction. Toward this end, Hampel and Garreis recently analyzed their usefulness in a thorough review [44], highlighting that HMGECs are a convenient tool for studying meibomian gland cell physiology, although limitations, especially regarding functions related to limited lipid production and hormonal influence, must be taken into account when drawing conclusions from studies performed with these cells.

The complex architecture of meibomian gland acini is crucial for the physiology of these glands and, therefore, conventional 2D cultures may be very limited in providing physiologically relevant results. For these reasons, there have been numerous attempts to establish 3D models using animal gland cells. The first 3D culture of meibomian glands was prepared in 2016 using rat cells [45]. In 2018, Asano et al. used HMGECs and different biomaterials and techniques to create 3D models of human meibomian glands [46]. First, they used different membranes (Millicell^®^-HA, Millicell^®^-PCF, and ThinCert™) as substrates on which HMGECs were seeded and cultured in air-lifted conditions. Millicell^®^ membranes were not appropriate to culture HMGECs but, on the contrary, when using ThinCert™, cells were able to create a multilayered epithelium. The authors also used highly porous scaffolds (Alvetex 3D scaffold membranes) in which cells were grown, first under submerged conditions and then in air-lifting conditions. In these 3D scaffolds, HMGECs differentiated into sphere-shaped colonies on the apical surface. Finally, the hanging drop technique, initially described by Harrison in 1907 and mentioned at the beginning of this review, was also used to establish 3D cultures of human meibomian glands [46]. Around 1000 cells were seeded in a 20 µL drop of proliferation medium on the inside of petri dish lids. The lids were then placed in their respective dishes containing PBS to generate a humidified atmosphere. Hanging drops were cultured in these conditions for 48 h, and cells exhibited the morphology of differentiating meibocytes, with lipid droplets in the cytoplasm and desmosomes connecting the cells.

A human meibomian gland ex vivo culture model was also described by Rötzer et al. [47]. They obtained upper eyelids from cadaveric donors and isolated tarsal plates that were subsequently sliced and placed in culture. Cells remained viable after 1-day cultivation, and the authors concluded that this model is a promising approach to study the pathophysiology of meibomian glands and to identify and test potential drugs to treat meibomian gland dysfunction.

The previously described in vitro models of meibomian glands involve only acinar cells. However, ducts are also important in the correct functioning of these glands. No human model developed to date has included meibomian gland ducts, although both acini and ducts were studied using an organotypic culture of mouse meibomian glands [48]. In this model, the authors cut tarsal plates into segments, plated them on Matrigel^®^, and kept them alive and functional for up to 72 h. With this model, the authors were able to show that after the administration of proinflammatory cytokine IL1β, acini cells accumulated lipids, and the ducts underwent keratinization. Thus, this model can be useful for studying the mechanism involved in meibomian gland dysfunction.

As discussed above, 3D models of meibomian glands were not available until very recently. We anticipate that these novel strategies, along with the new ones to come, will advance the knowledge of meibomian gland pathophysiology.

### 2.2. Cornea

The cornea is the outermost tissue of the anterior segment of the eye, and along with the tear film, constitutes the main barrier that topically administered drugs have to surmount [49]. For this reason, many in vitro corneal models are devoted to studying drug permeability and to performing barrier studies. The cornea is a complex tissue that contains five well-defined layers: the epithelium, Bowman’s layer, a highly organized stroma, Descemet’s membrane, and the endothelium [50,51]. The cornea lacks vascularization but possesses dense sensory innervation. Many authors have developed different 3D in vitro models of the different layers of the cornea, but they are primarily of the epithelium, stroma, and to a lesser extent, endothelium. As the number of corneal models is extremely high, we will mainly focus on those that use human primary cells in this review.

#### 2.2.1. Corneal Epithelium 3D Models

The epithelium is the outermost layer of the cornea and is, thus, the first to contact the external environment. It comprises 5–7 layers of stratified squamous epithelial cells: superficial cells, wing cells, and basal cells [52]. Although it only accounts for 10% of the whole corneal thickness, it represents the main tissue barrier for the permeation of hydrophilic drugs [49]. The barrier function is one of the key functional properties of the cornea. For all of these reasons, most of the available corneal cell culture models only represent this part of the tissue. Corneal epithelial 3D models are frequently used for three purposes: eye irritation tests, wound healing assays, and permeability and toxicity studies.

Four reconstructed human corneal-like epithelium models are included as validated reference methods in the 18 June 2019 version of the Organisation for Economic Cooperation and Development Test Guidelines (OECD TG) 492 for assessing eye irritation or serious eye damage: EpiOcular™ (MatTek, Ashland, MA USA), SkinEthic Human Corneal Epithelium (HCE)™ (SkinEthic, Lyon, France), LabCyte CORNEA-MODEL24 (Japan Tissue Engineering Co. Ltd., Miyakitadori, Aichi, Japan), and MCTT HCE™ [53]. EpiOcular™ was developed and commercialized by MatTek Corporation [54,55,56,57,58,59] and provides an alternative to animal testing to determine Draize scores. However, it is prepared with nonocular cells. Indeed, it consists of normal human epidermal keratinocytes that form a stratified epithelium similar to that of the cornea. This model is not further discussed here since the nonocular origin of the cells represents a significant flaw. On the contrary, SkinEthic HCE™ was established with immortalized human corneal epithelial cells [60,61,62,63]. It has the advantage of using corneal cells but the inconvenience of using a cell line instead of primary cells. To solve these issues, two additional models with human ocular cells were developed. The MCTT HCE™ model, published by Jung et al. in 2011 [64], is prepared with primary human limbal epithelial cells and cultured to form a multilayered, differentiated corneal epithelium model. The authors analyzed the expression of corneal cell markers as well as the barrier function by transepithelial electrical resistance (TEER) and concluded that the model faithfully represents human corneal epithelium, suggesting its use as a new alternative eye irritation test. Finally, the LabCyte CORNEA-MODEL24 may address some of the shortcomings of the EpiOcular™ and SkinEthic models since it is fabricated with primary human corneal cells [65,66,67]. This model has the three main corneal epithelial layers (superficial cell layer, wing cell layer, and basal layer) that express several corneal markers, namely, cytokeratin 3, mucins, and cell adhesion proteins; the superficial cells have microvilli and therefore resemble the architecture of native corneal epithelium. This model was validated for the performance of eye irritation tests in 2019 [67].

In 2002, Han et al. described a “bioengineered ocular surface” using fibrin as a scaffold and human corneal epithelial stem cells [68]. As stated by the authors, the fibrin gel is nontoxic and fully resorbable, and it provides a favorable matrix for epithelial cell growth and differentiation during wound repair. Corneal cells grown in fibrin gels expressed keratin 3, and some expressed keratin 19 as well. This is important because, in addition to their use as cell markers, keratins ensure the anchorage between epithelial cells and between the epithelium and the basement membrane. Although this bioengineered tissue may have some clinical applications because materials of human origin are used, it does not represent the whole corneal structure, making it less useful for in vitro studies.

More recently, MatTek Corporation provided a new alternative for in vitro testing, the EpiCorneal™ model [69]. This model presents several advantages compared with their older model, EpiOcular™, as actual human corneal epithelial cells are used. In this improved model, primary human corneal epithelial cells obtained from cadaveric donors are cultured in an air–liquid interface to promote cell stratification. The authors evaluated the corneal barrier function by measuring TEER, and on the basis of their promising results, they proposed using the model for drug delivery studies. In addition, they have used the model to evaluate oxidative stress in corneal injuries [70].

Another recent 3D corneal epithelial model is QoBur, presented by the authors as a “two-in-one model to determine ocular irritancy and barrier integrity” [71]. This model was created with normal human limbal cells that were expanded and then seeded on Transwell^®^ inserts. Once cells reached confluence, they were cultured in an air–liquid interface to improve stratification. This model does not seem to represent the three layers of corneal epithelium, but there is a clear difference between the basal cells that express the proliferation marker Ki67 and are undifferentiated vs. the superficial cells that are fully differentiated. The barrier function of the model was also studied using TEER measurements and expression analysis of different junction proteins. The TEER values showed that the barrier function increased over time, and after 7 days in culture, the values were very similar to those of the native cornea and higher than those of other in vitro models.

In addition to the layered structure of the cornea, other special characteristics of this tissue have been emulated in the most sophisticated models. For instance, the cornea has a special curvature, which is extremely relevant in some studies, such as those involving contact lenses. Bearing this in mind, Postnikoff et al. developed a curved, stratified in vitro model of human corneal epithelium using immortalized cells [72]. Cells were cultured in curved inserts coated with collagen type I, submerged for 7 days, and then cultured in air-lifted conditions for an additional 7 days to induce stratification. The model is composed of 3–5 layers of epithelial cells and is specially designed to assess the biocompatibility of contact lenses.

Several authors have reviewed the different models used to study drug toxicity and penetration through the corneal epithelium [73,74,75,76,77,78]. From these reviews, we can conclude that the number of available models is high. However, most of them are based on animal cells or cell lines [79]. Since we consider human models to be much more relevant and there is a wide variety of them available for studying the cornea, corneal models using animal cells are excluded from the current review. Nevertheless, readers are referred to the abovementioned reviews to read about these other models.

Just underneath the corneal epithelium is the Bowman’s layer, the next layer of the human cornea. This is an acellular, non-regenerating layer composed of collagen fibrils and has a physiological function that remains unclear [80]. It is likely that it is usually excluded from current in vitro models for these reasons.

#### 2.2.2. Corneal Stroma 3D Models

In addition to the epithelium, a variety of corneal stroma 3D models are described in the literature. The basic properties of these models, in which fibroblasts and the extracellular matrix are major components, were described by Guo et al. in 2007 and by Ren et al. in 2008 [81,82]. Human primary corneal fibroblasts were cultured in Transwell^®^ inserts in the presence of FBS and vitamin C and allowed to grow for up to 5 weeks. During that time, fibroblasts synthesized their own extracellular matrix. The constructs were similar to the developing mammalian stroma. The effects of vitamin C on fibroblasts include the induction of proliferation, reduction of cell contact inhibition (allowing for stratification [83]), and stimulation of the production and secretion of extracellular matrix components [81]. In a study published in 2010, human corneal fibroblasts were stimulated with vitamin C and TGFβ1 to develop a model that resembles human corneal fibrosis [84]. This model was also used to study keratoconus [85] and therapies against corneal diabetes [86]. Subsequently, Sharif et al. included nerves to study their interaction with fibroblasts [87].

With the help of 3D bioprinting techniques, Isaacson et al. recently developed corneal stroma equivalents [88]. They used sodium alginate and methacrylated type I collagen to prepare a wide variety of bioinks, and human corneal stromal cells were obtained from cadaveric tissue. An important advantage of this method is the reproduction of the curved geometry of the cornea.

#### 2.2.3. Corneal Endothelium 3D Models

The deepest layer of the cornea is the endothelium, which is essential for maintaining the health of the whole tissue as it controls membrane ion pumps and prevents corneal edema. Corneal endothelial cells do not divide since they are arrested in the G1 phase of the cell cycle [89,90] and, therefore, it is difficult to culture them in vitro and to develop a model of primary corneal endothelial cells. Nevertheless, a few models in the scientific literature include these cells. Very recently, Hutcheon et al. published the development of a 3D in vitro model to study corneal endothelial cell maturation [91]. The authors combined a stroma model with endothelial cells and showed the presence of Descemet’s membrane. Arnalich-Montiel et al. fabricated tissue-engineered endothelial grafts with the purpose of performing endothelial keratoplasty [92]. Human primary corneal endothelial cells were expanded in vitro on fibronectin-coated plates and then cultured on decellularized corneal stroma. In this case, the objective of this model is not to perform in vitro experiments but, if possible, to maintain them in culture for a few days. This may constitute an interesting option for deepening knowledge on corneal endothelium pathophysiology.

#### 2.2.4. Multilayer or Whole Cornea Models (Corneal Equivalents)

Some authors have established more complete models of the cornea that include several layers of this tissue or, in some cases, all layers to create whole cornea models, also referred to as corneal equivalents. The latter usually contain three cell types: epithelial cells, keratocytes, and endothelial cells.

**Figure 2:**
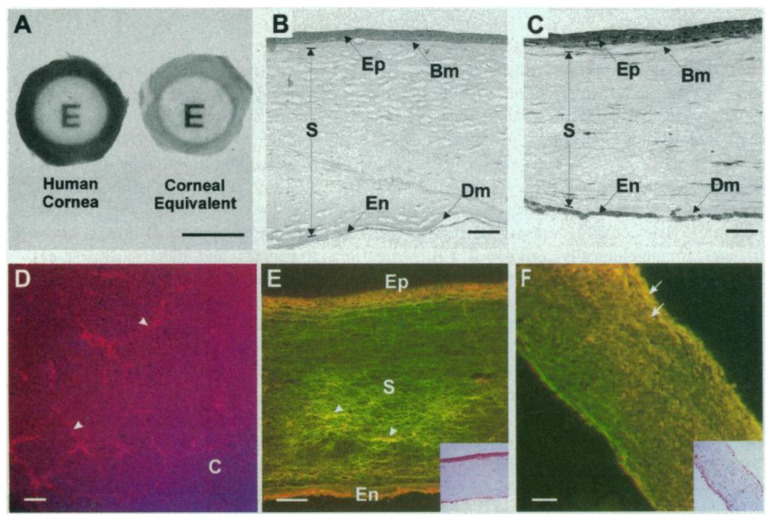
First three-dimensional model of the whole human cornea developed by Griffith et al. The corneal equivalents resembled human cornea in transparency ((**A**), scale bar: 10 mm) and histology (**B**,**C**). Histology of cultured human eye bank cornea (**B**) and corneal equivalent (**C**) show well-defined epithelial (Ep), stromal (S), and endothelial layers (En), and acellular limiting Bowman’s (Bm) and Descemet’s (Dm) layers. Scale bar: 100 µm. (**D**) By incorporating fibrin that supported angiogenesis, the authors produced a pseudo-sclera surrounding the corneal equivalents that contains microvessel-like structures (arrowheads). Scale bar: 500 µm. The corneal equivalents were different depending on the cell lines used (**E**,**F**). (**E**) In the most successful ones, prepared with cell lines with appropriate ion channel activities, epithelial (Ep) and endothelial (En) layers are distinct (red), and keratocytes (arrowheads) are present in the collagenous stromal matrix (S, green). Scale bar: 100 µm. (**F**) Corneal equivalents constructed with abnormal physiological function had in indistinguishable layers and transformed epithelial cells that invade the stroma (arrows). Scale bar: 100 µm. Reproduced with permission from [93], AAAS, 1999.

The first 3D model of the whole human cornea was reported by Griffith et al. in 1999. They developed a “morphological and functional equivalent of the human cornea” [93], proposed for use in biomedical research. They used immortalized cells derived from human corneal epithelial, stromal, and endothelial layers and a collagen–chondroitin sulfate substrate crosslinked with glutaraldehyde and treated with glycine as the matrix that represented the stroma. The constructs resembled the morphology and histology of cornea, and the physiological function was tested (Figure 2). Although it represented a remarkable milestone, the main limitation of the model was the use of cell lines that had to be screened and selected in order to avoid altered phenotypes.

Since then, several 3D corneal models using human primary cells to reproduce the three main layers of the cornea (epithelium, stroma, and endothelium) have been proposed as tools for in vitro research. A good example is the human tissue-engineered corneas produced by Proulx et al., who used human primary cells isolated from normal corneas that were unsuitable for transplantation and a method that they called the “self-assembly approach” [94]. In this approach, no exogenous material is used. Instead, stromal fibroblasts (both dermal and corneal) are induced to secrete their own extracellular matrix by using vitamin C. After 28–35 days, the fibroblasts produce thick sheets of extracellular matrix that are then superposed to create the stroma. Endothelial cells are then grown on top of the stroma for 2–7 days. Following this, limbal epithelial cells are seeded on the other side, and after several days of submerged culture, they are switched to air-lifting conditions to induce stratification. These tissue-engineered corneas resemble native corneas in many aspects, and the authors suggested their use for studying corneal pathologies, as well as for pharmacological and toxicological studies.

Alaminos’ group also bioengineered human artificial corneas using fibrin-agarose scaffolds [95,96,97,98]. Corneal keratocytes were cultured after immersion in the scaffolds, and epithelial cells grown from limbal explants were seeded on top. The stromal substitutes had a transparency similar to that of the human native cornea [97], and the epithelial cells cultured in the air–liquid interface were mature, stratified, and developed intercellular junctions [96].

The cornea is the most densely innervated tissue in the human body. The interactions between nerves and the stromal and epithelial cells are essential for maintaining the health of the ocular surface. Thus, the study of these interactions is paramount, and some in vitro models have been developed with the aim of reproducing this feature. In 2017, Wang et al. developed a novel 3D corneal model with epithelium, stroma, and innervation [99]. In this model, the authors used primary cells of both human (corneal epithelial and stromal cells) and animal (chicken dorsal root ganglion cells) origin, and silk films were used to reproduce the corneal stroma. The model was later validated for the study of nociception [100] and modified to model diabetic corneal neuropathy [101]. A step-by-step guide to preparing this 3D system was later published [102].

Notwithstanding the complexity of these corneal equivalents, their benefits are clear since the inclusion of more than one layer is important for measuring several parameters, such as eye irritancy. For instance, in a report from an Expert Meeting convened by the European Centre for the Validation of Alternative Methods in which experts analyzed different eye irritation tests, the authors concluded that the methods whose models include a stroma have the greatest potential to distinguish severe eye irritants from other toxic/damaging classes [103].

Another interesting approach is Ocular DynaMiTES [104]. To create this dynamic model, the developers used a formerly designed Dynamic Micro Tissue Engineering System (DynaMiTES) and applied it to the human hemicornea (HC) construct [105,106,107]. They used immortalized cells from human corneal epithelium (HCE-T cell line established by Araki-Sasaki [108]) and human corneal keratinocytes (HCK cell line [109]) to obtain a test platform for improved absorption studies of the anterior eye. Interestingly, the cells were cultured inversely, with the epithelial cells at the bottom of the insert and the keratinocytes above, separated by the insert membrane. This model was used to perform absorption and dynamic permeation studies. Under dynamic conditions, the model could better predict in vivo results and, thus, the authors proposed the use of this platform as an improvement of the standard in vitro drug testing procedures.

Corneal wounds can be produced by a variety of insults but are usually promptly repaired. However, in some conditions, repair does not occur, and the results can be as devastating as complete vision loss. Due to the great relevance of the proper healing of corneal wounds, the importance of its management, and the difficulties in developing treatments, corneal wounds are often studied in the laboratory [110]. The most typical model used to study wound healing is a simple scratch assay in a cell monolayer in culture [111]. This type of assay is easy to perform and interpret; however, it fails to accurately represent the real healing process that occurs in nature in stratified epithelia, such as those of the cornea. Thus, the development of 3D models that allow for the study of corneal wound healing would provide important advantages. Gonzalez-Andrades et al. established an in vitro model of the cornea with a barrier function that is useful for studying wound healing [112]. With the same purpose of assessing corneal wound healing, McKay et al. reported the development of a coculture system of human corneal epithelial cells and corneal stromal fibroblasts that secrete their own extracellular matrix [113].

Bioprinting techniques have been applied to corneal tissue engineering. Using optimized bioinks based on laminin and collagen, Sorkio et al. bioprinted different types of human corneal structures [114]. They used limbal epithelial stem cells to create a stratified corneal epithelium and used adipose tissue-derived mesenchymal stem cells to construct stromal structures. Cell viability and the expression of markers were promising after printing, and the constructs were successfully implanted into porcine corneal organ cultures. The 3D bioprinted constructs interacted with and attached to the host tissue after 7 days in culture.

In this section, we reviewed the main 3D cell culture models of the cornea that use human primary cells, which are summarized in Table 1. Other remarkable models that, instead, use immortalized cells, such as the human cornea construct (HCC) developed by Reichl et al. [105], the human hemicornea model developed by Engelke et al. [115], or the corneal model fabricated with collagen vitrigel membrane developed by Yamaguchi and Takezawa [116], are not further analyzed in this review. The use of some of the models presented in this section has allowed for improving the quality of in vitro research on the cornea.

### 2.3. Limbus

The limbus is a specialized region of the ocular surface that represents the boundary between the cornea and the conjunctiva. The main characteristic of the limbus is the presence of limbal epithelial and stromal stem cells that repopulate the corneal tissue. Readers may have noticed that some of the corneal models described in the previous section actually used limbal cells. They can typically be induced to differentiate into corneal epithelial cells by using different culture media. However, in this section, our aim is to describe in vitro cell culture models that faithfully represent the limbal structure.

Julie Daniels’ group developed a substrate based on collagen, the Real Architecture for 3D Tissue (RAFT), which was used to develop human limbal 3D models [117]. They cultured limbal fibroblasts in RAFT tissue equivalents and added limbal epithelial cells. Cultures were then maintained in air-lifting conditions. The fibroblasts were beneficial for the epithelial phenotype after air-lifting [118]. However, surprisingly, in another study, the authors observed that when tissue equivalents were cultured in non-air-lifted conditions, fibroblasts were not necessary [119]. They considered this as an important advantage to simplify regenerative medicine products. Thus, the employed approach depends on the objective of the study. For the purpose of conducting research in physiologically relevant models, we consider it more appropriate to use the complete model that includes fibroblasts.

A key functional property of the limbus is that it is located where limbal stem cells reside, and a special structure named the palisades of Vogt represents an ideal niche for these cells. Bearing this in mind, Daniels’ group also created bioengineered limbal crypts [120]. To do so, they prepared collagen hydrogels and removed liquid by using customized hydrophilic porous absorbers. The bases of these absorbers had microridges which allowed the researchers to create collagen constructs with a topography that resembled that of human limbal stromal crypts. The authors showed that the human corneal cell line HCE-T and human primary limbal cells were able to populate these constructs and to form multilayers. The superficial epithelium was characterized by large, squamous epithelial cells with no p63 expression (a putative stem cell marker), whereas cells that were on the crypts were small and had high expression of p63 and the proliferation marker Ki67. This indicates that after 3 weeks in culture, cells retained their proliferative capacity in the bioengineered limbal crypts.

The models described in this subsection represent prime examples of the importance of the 3D architecture to recapitulate physiological characteristics and functions.

### 2.4. Conjunctiva

The conjunctiva is the mucous tissue that extends from the limbus of the cornea to the inner side of the eyelids. It is composed of a stratified squamous nonkeratinized epithelium and an underlying loose stroma. In 1989, Steuhl described the presence of five types of epithelial cells in the conjunctiva [121]. Goblet cells are type I and highly specialized for mucin secretion. Cell types II–V are usually referred to as squamous stratified nongoblet cells and represent around 85–90% of conjunctival epithelial cells. Both goblet cells and stratified squamous cells secrete mucins, but only goblet cells can produce and secrete MUC5AC [122,123]. The secretion of mucins is one of the key functional properties of the conjunctiva and, thus, as in the case of cornea, most models of conjunctiva focus only on the epithelium. Nevertheless, it is surprising that in some of these models, cells do not produce MUC5AC. The conjunctival stroma is a loose connective tissue. The main cells that populate this stroma are fibroblasts, but blood vessels, lymphatics, and nerves are also present.

Conjunctival research has been neglected for a long time. Fortunately, the great importance of this tissue for whole ocular surface physiology has been recognized in the last 10 years, and the number of investigations in the conjunctiva has significantly increased during this period. Nevertheless, the number of models that represent the human conjunctiva is significantly lower than those of the cornea. There are two potential explanations for this reduced number of models. The first is related to the (traditional) lower interest in conjunctival physiology in comparison with that in the cornea. The other reason might be the difficulties encountered in culturing human conjunctival cells.

Cell cultures of primary human conjunctival cells have been successfully established since the 1990s. Diebold et al. developed and characterized human primary epithelial cultures from normal conjunctival biopsies obtained from patients who were undergoing cataract surgery [124,125]. These early examples of human primary cultures demonstrated a certain capacity for multistratification and in vitro production of a mucous-like material. Subsequently, Risse-Marsh et al. published a method to initiate and propagate primary human conjunctival epithelial cell cultures from biopsies under serum-free culture conditions [126]. Short-term cultures of conjunctival epithelial cells obtained by brush cytology were also established [127]. In 2003, Shatos et al. reported the isolation and characterization of cultured human goblet cells from conjunctival tissue obtained from patients undergoing periocular surgery [128]. This procedure has been widely used to study mucin secretion [129,130,131]. In 2013, we described an optimized protocol to culture human primary conjunctival epithelial and stromal cells from cadaveric donors [132] and used them to study ocular surface inflammation [132,133] and drug permeability [134]. Although these models are 2D, they provide a cell culture basis for developing novel 3D models.

Several publications refer to the expansion of conjunctival epithelial cells on top of different substrates to prepare conjunctival equivalents intended for transplantation into injured ocular surfaces. Eidet et al. reviewed conjunctival equivalents in 2015 [135]. The most commonly8 used substrate is the amniotic membrane [127,136,137]. However, because the use of biological material may present some inconvenience, other substrates, such as collagen [138,139,140,141,142], fibrin [143], poly(L-lactic acid-co-ε-caprolactone) [142,144], gelatin-chitosan [145], silk fibroin [144], or the cadaveric, acellular dermis AlloDerm^®^ [146], have also been used. Although these conjunctival equivalents have not yet been used as cell culture models, some of them have the potential to provide this function. However, further research is needed to determine whether they are useful for in vitro research. An interesting review on tissue engineering for conjunctival reconstruction was published by Schrader et al. in 2009 [147], in which the authors concluded that the minimum criteria that a conjunctival substitute must meet include flexibility and long-term stability, good tolerability, and the inclusion of epithelial layers that contain goblet cells. As they stated, the amniotic membrane meets some of these criteria and, indeed, it has been successfully transplanted into the ocular surface. However, new materials are also needed.

Multilayered cultures of conjunctival epithelium for use as in vitro models have also been developed using rabbit [148,149] and bovine cells [150]. However, these models only use animal cells, and the results must be interpreted with caution because of interspecies differences. A stratified culture of the human conjunctival epithelium using cells obtained from biopsies was established by Chung et al. [151]. When cultured in air-lifted conditions, the epithelial cells became stratified in up to 6–8 layers after 2 weeks of culturing. Mucin secretion, visualized with confocal microscopy after immune staining, was upregulated in response to the inflammatory cytokines TNFα and IFNγ. The authors concluded that this culture system could be used to investigate conjunctival epithelial cell biology and goblet cell differentiation.

Rosellini et al. used “an in vitro organ culture method to investigate human conjunctival epithelial basal precursor cells and their progeny within a more natural three-dimensional microenvironment” [152]. Conjunctival explants were cultured on gelatin sponges by following a method previously described for the cornea [153]. This organotypic model was maintained in culture for 14 days and developed a well-conserved 3D structure, which opens the possibility of using it for a wide variety of research purposes.

In a study published in 2014, Zorn-Kruppa et al. utilized two 3D models of human conjunctiva using the hTERT-immortalized human conjunctiva epithelial cell line HCjE [154] to examine conjunctiva-damaging substances [155]. The first model was a full conjunctiva model and included a collagenous stroma matrix containing corneal keratocytes. The second model lacked the collagen matrix (and was thus a conjunctiva epithelial model), and HCjE cells were directly plated in membrane inserts. In both cases, cells were cultured by submerging them for 2 days and then switched to air–liquid interface conditions for 6 days. Conjunctival cells became stratified (forming 4–8 layers) and were able to form a barrier. To confirm this, TEER was measured in the epithelial model, and claudin 1, ZO-1, and occludin were immunodetected in the full conjunctiva model. The authors found the tested chemicals had similar effects when comparing corneal and conjunctival models. They suggested the use of these models to investigate the influence of substances on barrier function or to answer basic biological questions.

Using a different approach, Fiorentzis et al. grew spheroids with the normal human conjunctival epithelial cell line HCjE-Gi [156]. In the same study, they also prepared spheroids with two conjunctival melanoma cell lines and compared the cytotoxic effects of different drugs obtained when using 3D spheroids or 2D models. Thus, this model can be used to study the cytotoxicity of different drugs.

**Figure 3:**
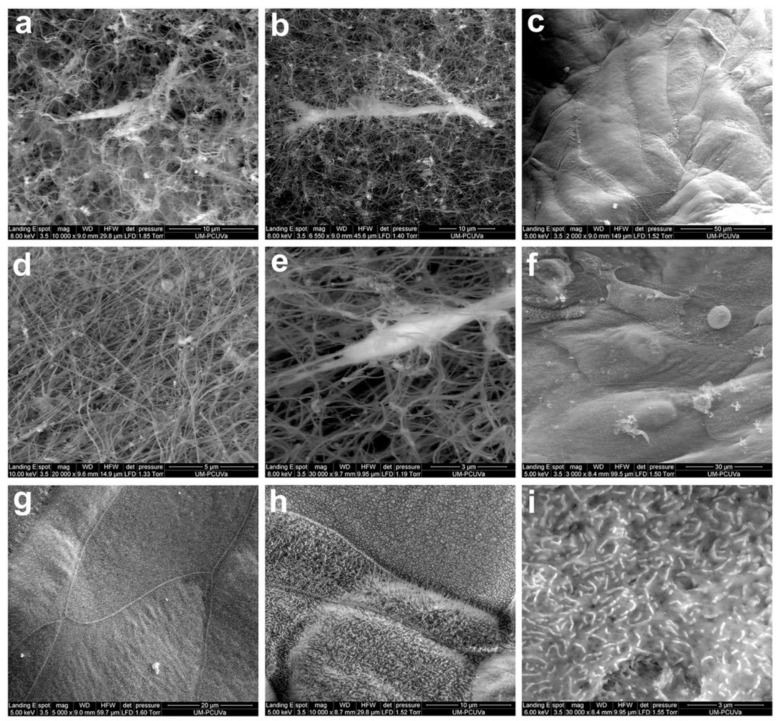

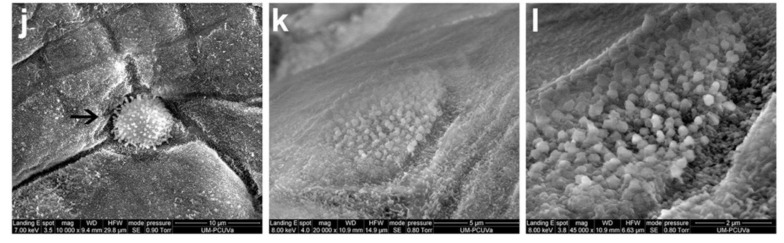
Scanning electron microscopy images of the three-dimensional model of human conjunctiva developed by García-Posadas et al. Fibrin used to prepare conjunctival equivalents was obtained from either human plasma (**a**–**c**) or cryoprecipitate (**d**–**f**). The main difference between them is that scaffolds prepared with cryoprecipitate showed a higher density of fibrils than those prepared with plasma. Fibrin scaffolds allowed the growth of fibroblasts inside them (**b**,**e**), and the growth of epithelial cells on top (**c**,**f**). The epithelial cells covered all the matrix surface, growing tightly adhered between them (**g**). In addition, the epithelial cells showed microvilli (**h**,**i**). It was possible to observed rounded cells ((**j**), arrow) and mucous granules (**k**,**l**) that suggest the presence of goblet cells in the 3D model. Reproduced from [157], PLoS ONE, 2017.

The main limitation of the conjunctival models described so far is that they either are single human cell monolayers [128,132] or use primary non-human animal cells [148,149,150] or human cell lines [155,156]. Chung et al. developed a 3D model using human primary cells; however, it only represents the epithelium and not the full-thickness conjunctiva [151]. Less attention has been paid to conjunctival stroma and their most abundant cells, the fibroblasts. To overcome these limitations, we developed a complete human conjunctival 3D model that was prepared with a fibrin-based scaffold and human primary cells [157]. The fibrin matrix represents the conjunctival stroma, and human fibroblasts were embedded in the scaffold (Figure 3). On top of the scaffold was a stratified epithelium that contained human conjunctival goblet cells. We showed that the production of both MUC5AC and the proinflammatory cytokine IL6 could be modulated by inducing changes in the conjunctival construct, thus allowing us to represent some of the typical features of common diseases such as allergic conjunctivitis or dry eye disease, increasing the application range of this model. In 1994, Tsai et al. reported a conjunctival equivalent in which human conjunctival epithelial cells were grown on collagen gels containing fibroblasts [158]. However, to the best of our knowledge, this model was not further studied or tested for any application. According to our literature review, these are the sole 3D models of the full-thickness conjunctiva using exclusively human primary cells.

Recently, Mitani et al. developed a 3D model of the conjunctiva containing rabbit primary conjunctival fibroblasts and a new human conjunctival epithelial cell line, called iHCjECs [159]. They used collagen gels as the scaffold, into which the fibroblasts were embedded. The epithelial cells became stratified on top of these scaffolds and expressed conjunctival markers such as CK13, CK19, and MUC5AC. The authors concluded that “iHCjECs may be a useful experimental cell line for experimental studies of the properties of conjunctival epithelial cells as well as for physiopathological or toxicological studies”, but this model has the drawback of using animal cells and cell lines.

In summary, there are several available 3D models of the conjunctiva. Different biomaterials have been used as scaffolds that represent the conjunctival stroma and/or allow for the culture and transport of epithelial cells. Besides the amniotic membrane, collagen in different forms is the most commonly used scaffold material. Some of the described models use human cell lines or animal cultures. However, since there are published protocols for culturing human primary conjunctival cells, it is possible to combine all of this know-how to create new 3D models using human primary cells that better represent the native conjunctiva.

### 2.5. Conjunctiva and Uvea in Ocular Cancer

The conjunctiva and the uveal structures (iris, ciliary body, and choroid) share an important feature for eye protection: they possess pigmented cells, namely, melanocytes. Melanocytes can undergo neoplastic transformation and give rise to melanocytic tumor lesions. Neoplastic melanocytes can be present not only in any of the uveal structures but also in the conjunctiva. Uveal melanoma is the most life-threatening cancer that affects pigmented ocular tissues. It has the particularity of being metastatic via the hematogenic route, especially in the liver, which, in most cases, markedly reduces patients’ lifespans. Conjunctival melanoma is not as aggressive, spreads to regional lymph nodes, and represents 2% of all ocular melanomas [160], but it is associated with a high mortality rate (25–30%) despite treatment [161].

This is a field of research in which in vitro experiments using cell monolayers have been crucial for gaining insights into the basic biology of the tumor and identifying potential targets to develop effective therapies. There are many examples of interesting investigations using human conjunctiva melanoma cell lines [162], human primary and metastatic uveal melanoma cell lines, and short-term primary cells [163]. However, the possibility of developing 3D models has gained strength, particularly in the last 10 years, which has been primarily driven by the scarcity of representative and well-characterized melanoma animal models [163]. Tumor cell spheroids that are produced from a single cell type or are multicellular, resembling micrometastases, appear to be useful in vitro tools for developing more complex functional assays for anticancer drug evaluation.

For instance, 3D uveal melanoma cultures using different uveal melanoma cell lines, such as OCM1 and C918, have been used to study resistance to herpes simplex virus type 1 (HSV-1)-mediated oncolysis as potential oncolytic therapy [164]. For the 3D culture, the authors used Matrigel^®^, a commercially available artificial extracellular matrix that is rich in laminin, which allowed invasion from melanoma cells either individually or after forming multicellular spheroids with different vasculogenic mimicry patterns according to their invasive potential and increased resistance to HSV-1.

Another example is the use of the 3D spheroid culture system for primary and metastatic uveal melanoma cell lines to evaluate the effect of electrochemotherapy, which is a combination of electroporation and chemotherapy [165]. The authors analyzed the cytotoxic effect of this form of chemotherapy on the 3D spheroids and determined its effectiveness as it elicited a significant reduction in tumor size and viability compared with electroporation or chemotherapy.

The same authors recently reported the development of 3D tumor organoids using conjunctival melanoma cell lines [156]. The authors used the human conjunctival melanoma cell lines CRMM1 and CRMM2 to form spheroids, in which they analyzed the cytotoxic effect of electrochemotherapy and other chemical treatments, such as 5-fluorouracil, mitomycin C, or bleomycin. Afterward, they published an automated image analysis method that can be used for the assessment of tumor growth and changes in the size of 3D tumor organoids following anticancer treatments [166].

Therefore, there are several interesting approaches to culture pigmented cells from conjunctival and uveal tissues, study their neoplastic transformation and metastasis mechanisms, and use them to test anticancer drugs.

### 2.6. Trabecular Meshwork

The trabecular meshwork (TM) is involved in the aqueous humor outflow, an essential biological process that maintains intraocular pressure (IOP) at physiological levels. This tissue is formed predominantly by endothelial-like cells in a specialized connective tissue. As the primary site of IOP regulation, TM has attracted a great deal of attention to study changes in IOP related to the neurodegenerative group of diseases collectively named glaucoma. According to the 2019 World Report on Vision, glaucoma is the second leading cause of blindness worldwide, and to date, IOP is the only known modifiable factor in the development of this disease. Since 1984, the main approach to studying the pharmacological modulation of TM function has been primary TM cell culture [167]. Subsequently, the development of a stable human TM cell line [168] and conventional 2D studies on tissue culture plastic have provided a valid model for pharmacological studies, although limitations remain in understanding the complexity of human TM biology.

In 2013, Torrejon et al. [169] published the first example of a 3D culture of primary human TM cells obtained from donor tissue rings that were discarded after penetrating keratoplasty. They were able to generate a 20 μm thick multilayered in vitro TM that retained characteristics that are relevant for its in vivo functions, such as extracellular matrix deposition, myocilin gene expression, and response to IOP-reducing agents. To achieve this functional 3D model, the authors used a highly porous membrane generated by photolithography as a scaffold with uniform pore size, shape, and beam width that provided topographical cues for the attachment of TM cells. This model was refined, a few years later, to recreate a 3D diseased (fibrotic) human TM model [170]. In this study, the authors used the same photolithography-generated scaffold for the primary TM cells, and after 14 days in culture plus 2 days in serum starvation, they exposed the 3D constructs to the steroid prednisolone acetate for 3, 6, or 9 days. The results of steroid exposure for 9 days revealed changes in the expression of several markers that are typically observed in patients with steroid-induced glaucoma, such as increased expression and secretion of myocilin, increased deposition of fibronectin and collagen IV in the extracellular matrix, intensified actin fiber crosslinking, decreased gene expression of metalloproteinases, increased gene expression of TGFβ, decreased phagocytosis, increased transmembrane resistance and, consequently, decreased outflow facility. Furthermore, the 3D constructs exhibited a response to IOP-reducing agents. Therefore, the bioengineered 3D human TM showed a functional response in vitro that resembled its behavior in steroid-induced glaucoma patients, and its usefulness for studying the physiopathology of the human outflow pathway and drug testing was demonstrated.

Another approach to achieving 3D TM cultures is to seed human TM cells in Matrigel^®^. Three-dimensional human TM cultures in Matrigel^®^ are responsive to different stimuli and situations, which is promising for advancing the understanding of glaucoma development. A recent example of an in vitro 3D human TM model using commercially available primary TM cells (ScienCell Research Laboratories) and Matrigel^®^ was published in 2017 [171]. Three-dimensional constructs were exposed to TGFβ2 and dexamethasone to induce morphological and functional changes, which were similar to the observations of Torrejon et al. in their steroid-exposed 3D in vitro TM model. The authors used this model to investigate the proinflammatory effect of benzalkonium chloride, which is the most commonly used preservative in topical anti-glaucomatous medication. Other authors [172] also cultured commercially available human TM cells (Cell APPLICATION INC.) in 3D Matrigel^®^ to study the effect of oxidative stress as this biological process is known to contribute to the development of glaucomatous disease [173].

A different material to support 3D human TM cell growth was reported by Waduthanthri et al. in 2019 [174]. In this paper, the authors reported the usefulness of a peptide hydrogel system (MAX8B) for bioengineering of a 3D TM scaffold. Human TM cells were obtained from the corneoscleral rim tissues of a healthy donor under ethical approval. The bioengineered 3D TM was responsive to dexamethasone treatment in terms of outflow facility. The authors considered their 3D system to be useful not only for drug testing but also as a potential injectable “TM implant” for complementary therapy in glaucoma patients because of its biocompatibility, as determined by human TM cell growth and proliferative capacity. An investigation of more complex matrices to support 3D TM cell growth for mechanistic studies is ongoing, and the focus is currently on understanding the complexity of the extracellular matrix microenvironment of human TM and its changes in glaucoma patients [175].

Finally, a recent paper identified a resident population of multipotent progenitor cells in the transition area between the periphery of the corneal endothelium and the anterior nonfiltering portion of the TM known as Schwalbe’s line [176]. These progenitor TM cells can be effectively isolated and expanded using a 3D culture system in Matrigel^®^. Progenitor TM cells need to be initially expanded in 2D culture using FBS-supplemented embryonic stem cell medium, and then cells from passage 2 can be seeded on 3D Matrigel^®^ to recover the expression of embryonic stem cell markers that were lost after the passage of primary cells [177]. This model is used to study TM alterations in glaucoma, and the long-term goal is to elucidate whether TM progenitor cells might be used for treating glaucoma or even corneal endothelial dysfunction, considering its ability to differentiate into corneal endothelial cells.

In summary, the human TM is quite a difficult tissue to model in either normal or diseased conditions. Nevertheless, there are interesting culture approaches that have shown great potential to increase the understanding of the differential TM response to normal and pathologic stimuli. In particular, the identification of progenitor TM cells holds promise for the development of a different strategy to treat glaucoma.

### 2.7. Lens

The lens has a prominent role in vision because its particular tissue structure enables visual accommodation throughout an organism’s life. The lens is transparent, possesses an important refractive index, and lacks vasculature. Morphologically, the human lens is composed of two cell types, namely, epithelial cells and fibers, enclosed by the lens capsule, which is actually a basement membrane. Epithelial cells proliferate and migrate and eventually differentiate into fiber cells, which are characterized by extensive elongation, structural specializations, and the accumulation of specific proteins such as crystallins. This remarkable process occurs throughout the organism’s lifespan, and alterations in the lens structure and, consequently, in its transparency can lead to blinding pathologies such as cataracts.

In addition to its application in developmental studies, the human lens draws considerable research interest for its involvement in cataract formation, and many efforts have been put into developing different study models. Currently, different alternatives are available to study lens pathophysiology and include the primary culture of human lens epithelial cells obtained from very young donor eyes, commercially available human lens epithelial cell lines, inducible pluripotent stem cells, and lentoid bodies [178]. Three-dimensional cultures can be obtained using primary cultures.

Primary pigment epithelial cells (PECs) from humans and other vertebrate species have the potential to transdifferentiate into the lens in vitro [179]. On the basis of this concept, Tsonis et al. [180] created a dedifferentiated human PEC cell line (H80HrPE-6) using primary retinal PECs from an 80-year-old person and established 3D cultures using hard agar or Matrigel^®^. The original primary cells were shown to possess the ability to transdifferentiate into the lens [181]. In these culture conditions, especially when cultured on Matrigel^®^, cells started to form aggregates that were able to form transparent structures resembling lentoids, in vitro, after just 4 days. These structures demonstrated significant crystallin synthesis and elongated morphological features, indicating that they were differentiated lentoid bodies. The authors considered this 3D in vitro system to be useful for studying not only the regulation of the transdifferentiation process, but also molecular aspects involved in lens differentiation and regeneration.

The human lens has been used in ex vivo organ culture for the biochemistry-related analysis of lens metabolism since the 1990s [182]. Usually, lenses without opacity were obtained 24 h post-mortem from cadaveric donors in eye tissue banks and maintained in DMEM or Eagle’s minimum essential culture medium supplemented with 10% fetal calf serum for short-term experiments. Another classical approach was to perform sham cataract operations on human donor eyes, placing the whole capsular bag ex vivo in culture to determine the resilience of cell proliferation [183]. This can also be achieved with a donor lens with an already-implanted intraocular lens [184]. The purpose of using such an ex vivo culture system was to advance the understanding of a clinically relevant situation, namely, the posterior capsule opacification that occurs in a significant proportion of patients after cataract surgery. These models take advantage of the fact that all capsular bags maintain a large population of viable cells on capsular surfaces with proliferative capacity many years after cataract surgery [185]. Capsular bags with or without implanted intraocular lenses can remain viable in culture for up to 100 days [184]. The main advantages of this model are the maintenance of tissue integrity while allowing for the study of the effects of molecular activators and inhibitors, morphological changes, and their correlation with gene and protein expression changes in the tissue. Some examples are changes in calcium signaling in response to different stimuli [186,187], the condition of lenses maintained in different culture media and buffers [188], and the inflammatory response upon TNFα treatment [189], among others.

The lens capsule has even been used as a matrix for the ex vivo expansion of limbal stem cells [190,191] or corneal endothelial cells [192] intended for ocular surface reconstruction.

Another example of a 3D human lens ex vivo model was reported in 2014 [189]. The authors cultured what they called human anterior lens capsule-lens epithelial cells (aLC-LECs), which were prepared by dissecting human lenses obtained from cataract surgeries. aLC-LECs explants were placed in a culture dish, with the concave side with the LECs on the top and oriented upwards. Then, the viscoelastic material HEALON OVD was placed on top of the explants for flattening purposes. The explants were maintained in culture from 6 to 48 days and, eventually, outgrowing epithelial cells migrated from the explant while maintaining a proliferative status. With this culture method, the authors were able to study changes in calcium signaling upon mechanical and acetylcholine stimulation and to observe TNFα-induced IL-8 secretion. An important limitation of this work is that even though the explant remained in culture for a long time, measurements were performed on the outgrowing LEC monolayer rather than on the explant itself, although LECs attached to aLC are metabolically quite active. Nevertheless, the model is useful for studying different biological and morphological aspects of human LECs. The same authors studied the ex vivo pluripotency and proliferative capacity of cultured LECs growing out and migrating from the human anterior portion of the lens capsule in the same culture conditions [193].

Donor eyes from an eye bank that were discarded for transplantation received open-sky cataract extraction and laser photolysis to mechanically remove the LECs from the anterior capsule and the capsular bag [194]. The purpose of this work was to determine the effectiveness of laser photolysis to prevent posterior capsule opacity formation. Lens capsules that were stabilized with a capsular tension ring implanted during the surgical procedure were then maintained in submerged culture conditions for 3 months. During that time LEC growth was monitored under transmission light microscopy to determine the area of LEC coverage over the posterior capsule surface. Pharmacological [195] or other treatments [196] were shown to prevent posterior capsule opacity formation using the ex vivo human capsular bag model.

Lentoid bodies (LBs) are in vitro 3D structures created from human embryonic stem cells (ESCs) or induced pluripotent stem cells (iPSCs) that exhibit biological lens characteristics. Currently, in vitro LB formation from human ESCs can be achieved following a three-step complex protocol that involves (1) incubation with a combination of growth factors, such as Noggin, BMP4, BMP7, and FGF2, followed by monitoring lens differentiation markers to confirm the presence of lens progenitors in culture; (2) expansion of the lens progenitor population; (3) incubation with other combinations of growth factors, such as Wnt3a and FGF2. The eventual differentiated lentoid formation is evident after day 35 in culture [178,197].

**Figure 4:**
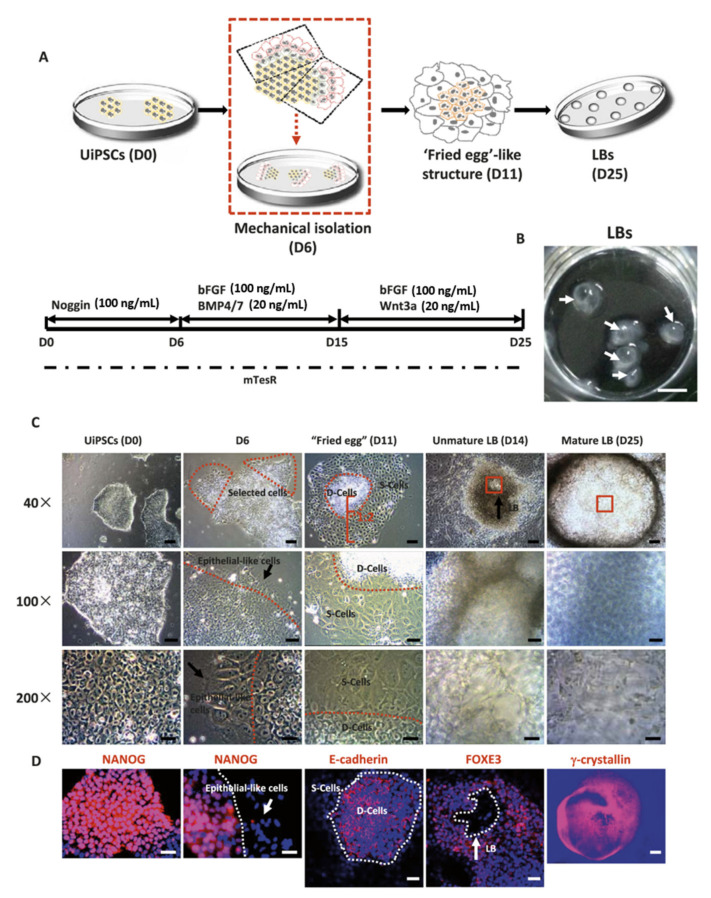
Differentiation of urinary human induce pluripotent stem cells (UiPSCs) into lentoid bodies (LBs) using the “fried egg” method developed by Fu et al. (**A**) Schematic diagram showing the stages of the “fried egg” method of LB generation. (**B**) Images of LBs after 25 days of differentiation. Scale bar: 3 mm. (**C**) UiPSCs at different days (**D**) in culture. “Fried egg” structure can be seen at day 11 (D11). The red square frame (D25) indicates a lens-like transparent structure. Scale bars: 100 μm (40×); 50 μm (100×); 30 μm (200×). (**D**) Immunofluorescence staining (red) of different markers. On day 0 the embryonic stem cell marker NANOG is present in all iPSCs, whereas its expression is lost in epithelial-like cells (white arrow) on day 6 (scale bar: 30 μm); expression of the lens epithelial cell marker E-cadherin on D11 in D-cells (scale bar: 50 μm); loss of lens epithelial cell marker FOXE3 expression on D14 in fiber-like cells (white dotted frame and arrow; scale bar: 50 μm), and expression of the lens marker γ-crystallin on D25 in mature LBs (scale bar: 400 μm). Reproduced from [199], Invest Ophthalmol Vis Sci, 2017.

An early example of in vitro LB formation is the coculture of human fetal lens epithelial cells and fibroblasts derived from the ciliary body [198]. In this work, the authors observed differentiating lentoid structures associated with fibroblasts. This observation was accompanied by the fact that the lentoid structures, but not the surrounding undifferentiated lens epithelial cells, contained lens fiber cell-specific proteins, alpha A-crystallin, beta B2-crystallin, and gamma S-crystallin, which indicated lens morphological differentiation. However, differentiated LBs did not exhibit any optical capacity. Recently, Fu et al. published a variation of Yang et al.’s published method, known as the “fried egg” method (Figure 4), which facilitates the development of transparent LBs [199]. Following the above-described three-phase method with minimal variations, LBs are differentiated from iPSCs generated from human urinary cells using four Yamanaka factors (UiPSs). The protocol leads to the formation of cell clusters around day 11 in culture with a typical “fried egg”-like appearance. Cell colonies with this structure consist of multiple E-cadherin+ differentiating cell layers in the center of the colony, which eventually differentiate into LBs, and a loose arrangement of E-cadherin-supporting cells at the periphery surrounding the differentiating cells. At day 25, mature LBs express lens-specific markers and also exhibit a transparent morphology. Qin et al. used this model to study age-related cataract formation [200]. They showed that mature LBs gradually lost their clear boundary and transparent morphology from day 25 onward and became completely cloudy on day 55. The gradual loss of transparency in cultured LBs mimics, to some extent, cataract development in human lenses. The authors studied different risk factors involved in cataract formation, including oxidative stress and UV radiation, to investigate their influence in the in vitro opacification process experienced by mature LBs. They found that LBs were damaged by UV exposure and could not be used for further experiments; however, H_2_O_2_ accelerated opacification and increased the crystallin protein aggregation in LBs, which suggests that this 3D system can be used to investigate human cataract formation.

## 3. Conclusions

The anterior segment of the eye possesses complex structures with relevant roles in maintaining ocular health and vision. As we presented in this review, significant advances have been made in the last 30 years to improve its study. The development of specific 3D in vitro models is one such advancement.

Each tissue that constitutes the anterior eye has unique characteristics and, accordingly, 3D models present different challenges. Some corneal models, for example, are transparent. A few others represent the curved anatomy of the tissue, and there are some corneal cell culture systems that include innervation. Other models have even achieved the form of micromasses of neoplastic cells to mimic an in vitro tumor. However, no single model includes all of these characteristics. In the case of the conjunctiva, to the best of our knowledge, there are no 3D models that include both of the essential characteristics of the tissue: vascularization and the presence of a conjunctival-associated lymphoid tissue. Similar limitations are found for the other anterior eye tissues. In the case of the sclera, there are not even human 3D models. Therefore, there is a promising niche for further research focused on creating new sophisticated cell culture models.

The main limitation to developing a 3D model with primary cells is typically the obtention of tissues, especially from humans. As we have observed in the different models reviewed in this article, these tissues can be collected from biopsies, surgeries, or cadaveric donors, but none of them are easily accessible. These constraints explain why, in some structures, current models use animal cells or human cell lines.

Three-dimensional models of the anterior eye have evolved over time, progressing from multilayered systems that are stratified using the air-lifted technique to much more complex constructs that include different cell types and biomaterials. The use of diverse approaches to maintain cells in 3D culture has also changed, from the first years when the use of animal feeder layers was typical to the present, when the use of animal-derived components is usually avoided.

The eye is a very complex organ in which different tissues are interconnected and work as a whole. For this reason, it would be incredibly valuable to study different tissues and their communication at the same time. In 2017, Lu et al. reported the establishment of an in vitro model of the ocular surface and tear film system [201]. The aim of this research was to study the pathogenesis of dry eye disease, and to this end, the authors cocultured rabbit conjunctival epithelial and lacrimal gland cells. They combined lacrimal gland spheroids with a stratified culture of conjunctival epithelial cells, including goblet cells. In addition, they exposed the model to proinflammatory cytokines to mimic dry eye disease conditions. This system provides a physiologically relevant model to study this pathology and is a good example of the combination of different tissues to develop more robust models. Unfortunately, there appears to be no similar approaches using human cells based on our survey of the literature. However, in this review, we discussed several models of the human cornea, conjunctiva, lens, etc., which may be combined to obtain a more sophisticated model of the anterior eye.

In this article, we reviewed the main 3D in vitro models that have been developed to study human anterior eye tissues. Any omission of relevant works in this field was not intended. Our aim is that readers find this review useful and will be encouraged to include some of these models in their studies and, furthermore, be inspired to combine these models and create new ones.

## Figures and Tables

**Table 1:** Summary of the main milestones achieved in corneal models developed with human primary cells.

Layer	Milestones	References
Epithelium	Validation of reconstructed human cornea-like epithelium (RhCE) models for eye irritation tests	[53,54,55,56,57,58,59,60,61,62,63,64,65,66,67]
	Representation of corneal curvature	[72]
Stroma	Development of models with fibroblasts that produce their own extracellular matrix	[81,82,83,84,85,86]
	Use of 3D bioprinting to develop corneal equivalents	[88]
Endothelium	Combination of a stromal model with endothelial cells and the presence of Descemet’s membrane	[91]
	Fabrication of tissue-engineered endothelial grafts using decellularized corneal stroma as scaffolds	[92]
Corneal equivalents	Development of the first 3D model of the whole human cornea	[93]
	3D corneal model using human primary cells and the “self-assembly approach” (no use of exogenous material)	[94]
	3D corneal model with epithelium, stroma, and innervation	[99,100,101,102]
	Application of dynamic systems to a corneal 3D model	[104]
	3D models of human corneal wound healing	[112,113]
	Application of bioprinting to create corneal constructs	[114]
